# Effect of tiger milk mushroom (*Lignosus rhinocerus*) supplementation on respiratory health, immunity and antioxidant status: an open-label prospective study

**DOI:** 10.1038/s41598-021-91256-6

**Published:** 2021-06-03

**Authors:** Eugenie Sin Sing Tan, Teik Kee Leo, Chung Keat Tan

**Affiliations:** 1 School of Healthy Aging, Medical Aesthetics and Regenerative Medicine, Faculty of Medicine and Health Science, UCSI University, Kuala Lumpur, Malaysia; 2 Research and Development Department, Nexus Wise Sdn Bhd, Selangor, Malaysia

**Keywords:** Respiration, Nutrition, Nutritional supplements

## Abstract

Tiger milk mushroom (TMM; *Lignosus rhinocerus*) have been used for a long time by indigenous communities in South East Asia regions as traditional medicine for different ailments, including respiratory disorders. The beneficial effects of TMM have been proven through in vivo and in vitro models, but these effects have yet to be validated in a clinical study. In this study, the beneficial effects of TMM supplementation were investigated in 50 voluntary participants. Participants were required to take 300 mg of TMM twice daily for three months. Level of interleukin 1β (IL-1β), interleukin 8 (IL-8), immunoglobulin A (IgA), total antioxidant capacity, malondialdehyde (MDA), 3-nitrotyrosine (3-NT), 8-hydroxydeoxyguanosine (8-OHdG), pulmonary function and respiratory symptoms were assessed during baseline and monthly follow-up visits. Results demonstrated that supplementation of TMM significantly (*p* < 0.05) suppressed the level of IL-1β, IL-8, MDA, as well as respiratory symptoms. In additional to that, TMM also significantly (*p* < 0.05) induced the level of IgA, total antioxidant capacity, as well as pulmonary function. Analyses of data indicated that gender and BMI were factors influencing the outcomes of antioxidant status. Collectively, our findings suggested that TMM supplementation effectively improves respiratory health, immunity and antioxidant status.

Tiger milk mushroom (TMM; *Lignosus rhinocerus*) was first mentioned in the “The Diary of John Evelyn” about 400 years ago. It is an important medical product which was received as repository’s collection by the Order at Paris from Jesuits of Japan and China^1^. This mushroom is commonly known as “cendawan susu rimau” or “kulat susu rimau” meaning “Tiger Milk mushroom”. TMM was successfully cultivated in 2009; thus, making it commercially available and spurring researches on its therapeutic uses. Its sclerotium extracts had been shown to be antioxidant^2^, antimicrobial^3^, anti-inflammatory^4^, anti-asthmatic^5^, and able to enhance immunomodulatory activities^6^. Recent in silico absorption, distribution, metabolism, excretion, and toxicity (ADMET) analysis revealed that most compounds (31 from 36 compounds) from TMM extracts were orally active, and high absorption rates were demonstrated by at least ten of the compounds^7^. Along with that, a preclinical toxicology study also showed that there was no treatment-related sub-acute toxicity following 28-days oral administration of 1000 mg/kg TMM. The no-observed-adverse-effect-level (NOAEL) dose was higher than 1000 mg/kg^8^.

Recently, World Health Organisation (WHO) had stressed on the importance of respiratory health and protecting the vulnerable lung from external stressors such as particles, chemicals and infectious organisms^9^. Respiratory diseases such as chronic obstructive pulmonary disease (COPD), asthma, acute lower respiratory tract infections, tuberculosis and lung cancer represents global health burden^10^. Upon exposure to external stressor, human’s first-line filtration involves nasal vibrissae, mucociliary escalator and cough reflex. Salivary immunoglobulin A (IgA) is an immunity defence at mucosal surfaces protecting against pathogens and smaller particles. C-terminal tail of IgA molecules disrupts cell-surface attachment of enveloped viruses which uses sialic acid as receptors such as the influenza A virus^11^. The potential role of secretory IgA in fighting severe acute respiratory syndrome coronavirus 2 (SARS-CoV-2) has also been highlighted in recent studies, preventing them from binding to epithelial cells^12,13^. Salivary IgA is said to be a predictor of upper respiratory tract infections (URTI); of which risk of URTI increases with decreased load of salivary IgA^14^.

Airway inflammation is body’s natural defence mechanism. In the lung, toll-like receptors (TLRs) activates inflammatory cells to produce growth, chemokines and pro-inflammatory cytokines such as tumour necrosis factor alpha (TNF-α), interleukin 1β (IL-1β), and interleukin 8 (IL-8). These cytokines evokes the neutrophils to migrate out of pulmonary capillaries into air spaces and performs phagocytosis^15^. As the lung is an vital organ responsible for providing oxygen, excessive inflammation can be detrimental to human health and it calls for need to establish lung homeostasis^16^. Anti-inflammatory properties of TMM was investigated in Sprague Dawley rats and results reported strong inhibitory effects on TNF-α production in macrophage cells^4^. To add, extracts of TMM also proven to be effective in reducing the infiltration of eosinophil into lungs of ovalbumin (OVA)-sensitized asthmatic rats. Thus, suggesting TMM as alternative treatment for acute asthma and to reduce airway inflammation^5^.

Respiratory diseases like asthma and COPD are also linked to oxidative stress^17^. Reactive oxygen species (ROS) formed damages cellular molecules, causing cell injury, and could induce respiratory cell death. Modulation of oxidative stress can aid in therapeutic management and prevention of disease^18^. Several studies had associated respiratory diseases with markers of oxidative stress such as malondialdehyde (MDA) for lipid damage, 8-hydroxy-2’-deoxyguanosine (8-OHdG) for DNA damage and 3-Nitrotyrosine for protein damage^19,20^. Elevated levels of markers had been reported for elderly and smokers with COPD as well as allergic asthmatic children^21–23^. These markers of oxidative stress had been proposed to be biomarkers of respiratory diseases; such MDA for asthma monitoring^22^, 8-OHdG for COPD and lung cancer^24,25^ as well as 3-NT for acute respiratory distress syndrome (ARDS) and viral infection^26^. Alongside, the Nasal Symptom Questionnaire (NSQ) is another useful clinical utility for scoring of nasal symptoms and allergic rhinitis^27^.

Indigenous communities in South East Asia regions traditionally used TMM for improvement of respiratory health and yet is known about its efficacy. As such, this study aims to evaluate effects of TMM on improvements of respiratory health utilising various indicators such as immunity, symptoms and oxidative stress.

## Results

### Characteristics of participant

**Figure 1:**
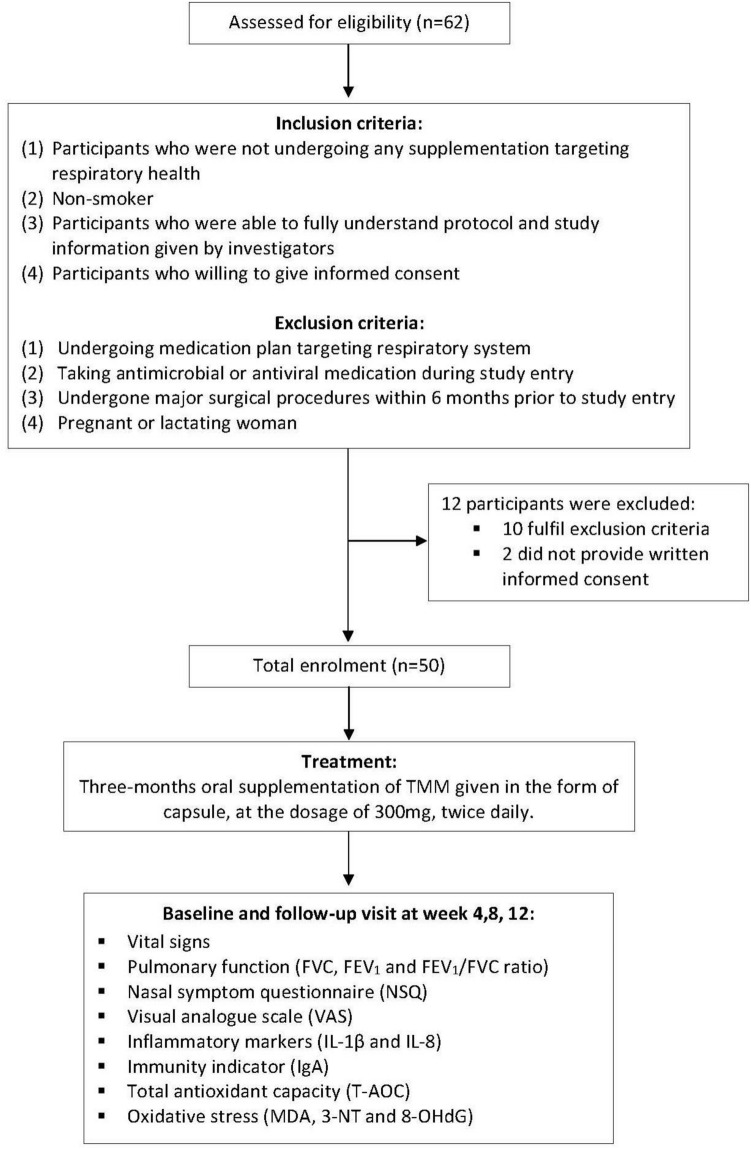
CONSORT protocol for the study described with flowchart.

Fifty participants with no background of chronic respiratory disease, non-smokers and not undergoing any supplementation targeting respiratory health have been recruited into this study. No dropout during follow up period (Fig. 1). Most participants were at the age of 30–35 years old (n = 27, 54%); followed by 41–45 years old (n = 14, 28%), 46–50 years old (n = 5, 10%) and lastly 36–40 years old (n = 4, 8%). There were more male participants (n = 28, 56%) as compared to female participants (n = 22, 44%). The majority of the participants were in the category of normal weight (n = 30, 60%); followed by overweight (n = 12, 40%), lastly were underweight and obese with 10% and 6% respectively (Table 1).

**Table 1:** Characteristics of participant.

Vital sign	Baseline	First follow-up	Second follow-up	Third follow-up	^a^p-value
**Blood pressure (mmHg)**					
Systolic	122.66 ± 20.62	120.52 ± 21.80	121.86 ± 16.56	123.25 ± 18.10	0.546
Diastolic	82.28 ± 13.56	81.24 ± 12.33	80.38 ± 13.26	82.34 ± 13.70	0.410
Heart rate (BPM)	80.8 ± 13.6	84.6 ± 10.7	81.6 ± 11.5	81.1 ± 11.1	0.124
Temperature (^o^C)	36.64 ± 0.39	36.74 ± 0.27	36.75 ± 0.24	36.80 ± 0.22	0.178

### Changes in vital signs

All the 3 vital signs showed no significant changes throughout the whole study (Table 2). Blood pressure of participant was within normal range, even after taking into account of the standard deviation. Variation of blood pressure between each visit was within 5 mmHg, which returned to be statistically not significant. Similarly, heart rate and temperature of participants were also showing very minimal changes between each visit.

**Table 2:** Changes in vital sign of participant during study.

Characteristic	Frequency
**Gender (n/%)**	
Male Female	28 (56.0) 22 (44.0)
**Age (years) (n/%)**	
30–35 36–40 41–45 46–50	27 (54.0) 4 (8.0) 14 (28.0) 5 (10.0)
**Body mass index (BMI) (n/%)**	
Underweight (< 18.5) Normal weight (18.5–24.9) Overweight (25–29.9) Obese (≥ 30)	5 (10.0) 30 (60.0) 12 (24.0) 3 (6.0)

*mmHg* millimetre of mercury; *BPM* beats per minute; ^*o*^*C* Celsius.

Values were expressed as means ± SD.

Statistically significant p values are marked in asterisks (*).

^a^p-value was calculated using general linear model (GLM) for repeated measures model, with sampling time point as within-subjects factor.

### Changes in respiratory health and immunity

Almost all of the respiratory health parameters showed significant changes (*p* < 0.05) after three-months supplementation of TMM, except FVC (Table 3). FVC changes were insignificant with some fluctuations in between the visits. On the other hand, FEV_1_ showed significant changes (*p* < 0.01) with 19.7% improvement by the end of study. Together with that, ratio of FEV_1_ to FVC was also significantly (*p* < 0.001) improved from 67.40 ± 19.76 to 85.73 ± 13.82, which is equivalent to 27.2% improvement. Respiratory symptoms as assessed by NSQ showed significant (*p* < 0.001) changes by the end of study; scoring was decreased from 8.96 ± 4.89 to 2.32 ± 0.79, equivalent to 74.1% of drastic changes. Likewise, VAS score as self-administrated by participants was also showing significant reduction (*p* < 0.001) from 2.54 ± 1.54 to 0.68 ± 0.49. Results showed that inflammatory condition of participant’s respiratory system was significantly (*p* < 0.001) improved after supplementation. Reduction in IL-1ß and IL-8 content were 54.9% and 40.8% respectively. IgA level increased from 4.83 ± 0.28 ng/mL to 10.26 ± 1.79 ng/mL, which reflected a significant (*p* < 0.01) improvement in respiratory immunity with the supplementation of TMM. Results doesn’t showed any significant differences when gender, age and BMI were being tested as between-subject effect.

**Table 3:** Changes in respiratory health and immunity of participant during study as indicated by clinical assessment and laboratory outcomes. IL-1ß and Il-8 were analysed from nasal lavage sample, IgA was analysed from salivary sample.

Parameters	Baseline	First follow-up	Second follow-up	Third follow-up	^a^p-value	^b^p-value	^c^p-value	^d^p-value
**Pulmonary function**								
FVC, mean ± SD FEV_1_, mean ± SD FEV_1_/FVC ratio, mean ± SD	5.75 ± 2.28 3.50 ± 0.96 67.40 ± 19.76	5.07 ± 1.94 3.60 ± 1.07 75.81 ± 18.72	5.12 ± 2.16 3.97 ± 1.39 82.05 ± 16.98	4.91 ± 1.92 4.19 ± 1.74 85.73 ± 13.82	0.217 < 0.01* < 0.001*	0.218 0.211 0.354	0.217 0.109 0.493	0.217 0.065 0.475
**Respiratory symptoms**								
NSQ, mean ± SD VAS, mean ± SD	8.96 ± 4.89 2.54 ± 1.54	5.96 ± 3.81 1.62 ± 0.89	3.96 ± 1.19 1.12 ± 0.67	2.32 ± 0.79 0.68 ± 0.49	< 0.001* < 0.001*	0.145 0.475	0.215 0.096	0.401 0.643
**Inflammation**								
IL-1ß (pg/mL), median (IQR) IL-8 (pg/mL), median (IQR)	3.38 (2.99–3.99) 15.27 (13.10–18.49)	3.06 (2.86–3.40) 13.69 (11.15–16.55)	1.70 (1.48–2.42) 10.15 (8.73–12.38)	1.49 (1.27–1.92) 9.33 (7.50–10.81)	< 0.001* < 0.001*	0.111 0.549	0.267 0.083	0.214 0.192
**Immunity**								
IgA (ng/mL), mean ± SD	4.83 ± 0.28	5.05 ± 1.30	7.06 ± 1.54	10.26 ± 1.79	< 0.01*	0.590	0.100	0.697

*FVC* Force Vital Capacity; *FEV*_*1*_ Forced Expiratory Volume; *NSQ* Nasal Symptoms Questionnaire; *VAS* Visual Analogue Scale; *IL-1ß* Interleukin 1ß; *IL-8* Interleukin 8; *IgA* Immunoglobulin A.

Statistically significant p values are marked in asterisks (*).

^a^p-value was calculated using general linear model (GLM) for repeated measures model, with sampling time point as within-subjects factor.

^b^p-value was calculated using general linear model (GLM), with gender tested as between-subject effect.

^c^p-value was calculated using general linear model (GLM), with age tested as between-subject effect.

^b^p-value was calculated using general linear model (GLM), with BMI tested as between-subject effect.

### Changes in antioxidant status of respiratory system

Results showed that total antioxidant capacity of participant was significantly (*p* < 0.001) increased from 0.703 ± 0.321 mmol/L during baseline visit to 1.188 ± 0.525 mmol/L in last follow up visit, which is equivalent to 68.9% of improvement. MDA content as the indicator of lipid peroxidation due to oxidative damage was significantly reduced (*p* < 0.001) from 1.10 ± 0.87 nmol/L to 0.34 ± 0.18 nmol/L, equivalent to 68.3% reduction. In contrast, 3-NT and 8-OHdG content as indicator of oxidative damage on protein and DNA were not affected with the supplementation of TMM (Table 4). When demographic factors were tested as between-subject effect, it was found that gender and BMI played a role in influencing the outcome of antioxidant status. Male was more responsive to supplementation as compared to female in increasing total antioxidant capacity. Result also showed that normal weight participant was statistically more responsive in suppressing production of MDA as compared to other categories of BMI.

**Table 4:** Changes in antioxidant status of participant during study as indicated by laboratory outcomes, antioxidant status was analysed from salivary sample.

Parameters	Baseline	First follow-up	Second follow-up	Third follow-up	^a^p-value	^b^p-value	^c^p-value	^d^p-value
**Antioxidant status**								
TAC (mmol/L), means ± SD MDA (nmol/L), median (IQR) 3-NT (ng/mL), median (IQR) 8-OHdG (ng/mL), median (IQR)	0.703 ± 0.321 0.94 (0.81–1.21) 0.50 (0.38–0.61) 0.91 (0.58–1.05)	0.867 ± 0.426 1.03 (0.91–1.22) 0.54 (0.44–0.69) 0.70 (0.23–0.98)	0.986 ± 0.360 0.40 (0.28–0.76) 0.29 (0.19–0.45) 0.55 (0.12–0.72)	1.188 ± 0.525 0.28 (0.26–0.33) 0.26 (0.18–0.37) 0.47 (0.13–0.77)	< 0.001* < 0.001* 0.183 0.139	< 0.05* 0.083 0.378 0.110	0.467 0.480 0.627 0.417	0.381 < 0.01* 0.549 0.609

*TAC* Total Antioxidant Capacity; *MDA* Malondialdehyde; *3-NT* 3-Nitrotyrosine; *8-OHdG* 8-hydroxy-2-deoxyguanosine.

Statistically significant p values are marked in asterisks (*).

^a^p-value was calculated using general linear model (GLM) for repeated measures model, with sampling time point as within-subjects factor.

^b^p-value was calculated using general linear model (GLM), with gender tested as between-subject effect.

^c^p-value was calculated using general linear model (GLM), with age tested as between-subject effect.

^b^p-value was calculated using general linear model (GLM), with BMI tested as between-subject effect.

## Discussion

This study is the first to report the beneficial effects of TMM on respiratory health through clinical approach. Our findings revealed that TMM supplementation is effective in improving overall respiratory health and immunity of the participants. Few parameters being assessed as the indicator of respiratory health has shown a significant improvement by the end of this study. Those parameters included pulmonary function, respiratory symptoms, VAS, interleukin level and immunoglobulin level. Ratio of FEV_1_ to FVC that reflects pulmonary function has shown 27.2% improvement after three months of TMM supplementation. Meanwhile, respiratory symptoms as assessed by NSQ and VAS have shown a drastic drop by the end of study, with the reduction of 74.1% and 73.2% respectively. The underlying mechanism responsible for such changes was likely due to the dropped in IL-1ß and IL-8 level, with 54.9% and 40.8% reduction respectively in this study. IL-1ß and IL-8 are both the major cytokines involved in the initiation and persistence of inflammation in airway and lung^28,29^. IL-1ß is an inducible cytokine and generally only expressed in damaged cells or triggered by pathogen product through the activation of pattern recognition receptors (PRRs)^30^. In the lung, IL-1β is produced by epithelial cells, alveolar macrophages, and mast cells and is notably upregulated in patients with respiratory disease^31^. IL-1β has been demonstrated to be closely associated inflammatory response causes progressive tissue fibrosis in lung^32^. IL-8 on the other hand is a secretory product produced mainly by stimulated macrophages. Its expression in monocytes can also be up-regulated by IL-1β^33^.

Previous studies have showed that TMM exerted the effect of modulating inflammatory properties in both in vivo and in vitro studies^4–6,34^. In vivo study showed that methanol extracts of TMM exhibits acute anti-inflammatory activities, by using carrageenan-induced paw edema test^4^. These anti-inflammatory activities were likely attributed by high-molecular-weight protein in TMM which exerts inhibitory effect on lipopolysaccharide (LPS)-tumour necrosis factor (TNF)-α production^4^. TNF-α was reported to be the key modulator that recruits inflammatory cells, stimulating the generation of inflammatory mediators such as IL-1ß and IL-8, increasing oxidative stress, and inducing airway hyperresponsiveness^35^. Apart from inhibitory action against TNF-α production, TMM also exhibited regulatory effect against macrophages^4^. The regulatory effect of TMM was suggested to be attributed by its high linoleic acid content. Linoleic acid inhibits inflammatory responses from macrophage through inactivation of nuclear factor (NF)-kappaB and activator protein-1 (AP-1) by suppressing oxidative stress and signal transduction pathway of signal-regulated kinase (ERK) and c-Jun N-terminal kinase (JNK)-1^36^. In addition, oral administration of linoleic acid was found to effectively reduced the autoinduction process of IL-1ß, as well as pro-IL-1ß gene expression^37^.Although macrophages played a critical role in immune function, but study showed that macrophage dysfunction in many respiratory diseases is highly prevalent^38^. Both IL-1β and IL-8 have been characterized as key contributors to the pathogenesis of many inflammatory lung diseases, including the bronchiectasis, pulmonary fibrosis, acute respiratory distress syndrome, chronic obstructive pulmonary disease and asthma^29,39^. The suppressing effect of TMM on overproduction of IL-1β and IL-8 is crucially important to conserve the lungs’ function and to prevent any damage from pro-inflammatory cytokines^40^. The improved pulmonary function, NSQ and VAS scoring from our findings have further validated that. Recent study suggested that any treatment targeting cytokine could potentially be an evolving treatment strategy in chronic inflammatory airway diseases^15^.

NSQ is a simple and accurate scoring system developed specifically to evaluate the symptoms caused by sinonasal diseases, such as sneezing, nasal obstruction, nasal discharge, olfactory loss, cough and quality of life^27^. The prevalence of chronic rhinosinusitis (CRS), most common type of sinonasal disease was recorded to be 5.5–19.9% in a few population-based studies conducted in the past 10 years and it is in an inclining trend^41,42^. Sinonasal disease was mainly triggered by persistent or recurrent episodes of infection or inflammation of one or both sinus cavities^43^. Thus, the significant improvement in NSQ scoring was most likely attributed by the anti-inflammatory effect of TMM supplementation. In addition to that, the antiproliferative effect of TMM could also contributed the improvement in sinonasal symptoms^44^. VAS is a psychometric scale that proven to be useful in recording sinonasal symptom severity^45^. Finding from VAS was well correlated with NSQ outcome, both of the scales showed similar degree of reduction in the end of study. Impaired peak lung function has been highlighted as one of the risk factor that contributed to development of chronic lung disease^46^. Low FEV_1_ and FEV_1_/FVC between 18 and 30 years of age predicted airflow obstruction in 20 years later, and that prediction is independent from smoking status^47^. In addition to that, impaired pulmonary function in early adulthood also leads to other health consequences in later life^48^. As such, TMM supplementation that can effectively improve pulmonary function suggested a preventive effect against development of pulmonary diseases. The protective effect of TMM supplementation could be attributed by its anti-inflammatory^34^, and immunomodulatory properties^6^. This finding also concurred with a recent study that reported airway relaxation effects of TMM^49^.

Mucosal membranes are critical as the first line of immunity defence in respiratory tract in preventing the invasion of pathogens through epithelial barrier^50^. IgA being the most predominant immunoglobulin isotype in mucosal tissue played its part through three main mechanisms; immune exclusion, intracellular neutralization and virus excretion^51^. In this study, IgA level of participants was doubled after three months of TMM supplementation, suggesting a strengthen respiratory immunity. Low IgA level is often being associated with increased risk of developing respiratory tract infection^52^. Although the underlying mechanism of IgA induction by TMM still remains unclear, but study done on *Ganoderma lucidum*, type of mushroom with close evolutionary relationship with TMM, have proved that IgA can be induced through the up-regulation of Toll-like receptor 4 (TLR4) dependent pathway^53^. Interestingly, the same study also reported that natural immunity can be activated without promoting inflammation, which is concurred with our present findings. Another study on medicinal mushroom highlighted that proteoglycan, which is a common constituents of medicinal mushroom enhanced the secretion of mucosal IgA by up-regulating the expression of polymeric immunoglobulin receptor (pIgR)^54^. PlgR had a dual role of transporting locally produced IgA across mucosal epithelial, as well as being the precursor for production of glycoprotein which improved immune functions of IgA^55^. As the first liner in immunity defence, enhanced production of IgA by TMM supplementation is important to prevent colonization of respiratory tract by pathogens and penetration of antigens through epithelial cells. The antiviral properties of IgA were well established by studies on Sendai virus, influenza virus, and rotavirus^51^. Most recent studies also suggested the potential role of IgA in fighting SARS-CoV-2 infection^12,13^.

Free radicals were generated in every single second and majority of them were generated as by-product from respiration process. In other words, respiratory system is particularly more susceptible to oxidative stress-mediated injury; mainly due to high endogenous oxygen concentration in lung and inhalation of exogenous air pollutants. Antioxidant as therapeutic alternatives to improve respiratory disease has gained much attention lately due to its easy accessibility through dietary intake. Recent review pointed out that although definitive data is still lacking, but available evidences suggested that dietary intake of antioxidant is closely associated with better pulmonary function, less lung function decline and reduced risk of COPD^56^. Our result indicated that three months of TMM supplementation can effectively increase total antioxidant capacity by almost 70%. This observation is presumably due to the high phenolic content in TMM^57^. Although a study showed that other non-phenolic compounds in TMM may possess the ability to scavenge radicals, but these compounds are yet to be identified warranting further studies for validation^58^. Epidemiological studies and associated meta-analyses had suggested long term consumption of diets rich in plant polyphenols to significantly improve antioxidant capacity and to offer protection against cancers, cardiovascular diseases, respiratory diseases and neurodegenerative diseases^59^. High polyphenol diet was associated with better pulmonary function in population based study^60^. In addition, TMM supplementation could potentially exert a protective effect against inflammation-related respiratory diseases as polyphenols had been proven to effectively reduce inflammation, by (1) up-regulating antioxidant gene expression, (2) attenuating endoplasmic reticulum stress signalling, (3) blocking pro-inflammatory cytokines, (4) suppressing inflammatory gene expression by stimulating histone deacetylase activity, or (5) activating transcription factors that antagonize chronic inflammation^61^. It is worth noting that male participants in our study is showing more significant changes in antioxidant capacity as compared to female participants. Similar observation has been discussed previously in view of the differences in the level of oxidative stress between male and female^62^.

Lipid peroxidation was found to be positively associated airway inflammation; reactive oxygen species (ROS) were generated by several inflammatory cells that participate in airway inflammation and their production will in turns amplify the inflammation in airway, thus created a positive looping^63^. MDA is the main product of lipid peroxidation often being used as biomarker to assess oxidative-mediated lipid damage. Its correlation with pulmonary function and respiratory disease severity was well established^64^. Our result indicated TMM supplementation exerted a suppressive effect on MDA production, which implicated its potential protective effect against oxidative damage. Such observation was likely attributed by improved total antioxidant capacity of participants. Plant phenolics has similar antioxidant activity as vitamin E with potentially higher potency than vitamin E to inhibiting lipid peroxidation, as well as scavenging superoxide anion and hydroxyl radicals^65^. Similarly, oral administration of TMM in diabetic animal model also found that elevated glutathione (GSH) level, superoxide dismutase (SOD), and catalase (CAT) activities to be associated with reduced lipid peroxidation^66^. Reduced lipid peroxidation is important to prevent many detrimental effects on airway function which were caused by oxidative stress, including airway hyperresponsiveness^67^, mucus hypersecretion^68^, epithelial shedding^69^, airway smooth muscle contraction^70^, vascular exudation^71^, and increased vascular permeability^72^. Our result also revealed that participants with normal BMI responded better to the supplementation as compared to other categories of BMI. This is in agreement with previous finding that pointed out greater efforts were needed to lower oxidative stress in obese patients, due to their excessive production of superoxide anion in the mitochondrial electron transport chain, as promoted by elevated plasma free fatty acids (FFA)^73^. In addition to that, studies also showed that with increasing BMI, there are greater levels of airway oxidative stress biomarkers due to induced inflammatory response associated with leptin, which eventually lowered corticosteroid responsiveness in asthmatic patients^74^. Although our study doesn’t observe any significant changes in 3-NT and 8-OHdG levels, but still charted a noticeable reduction of 50.67% and 48.29% respectively, suggesting a longer duration of study is required to monitor the long terms effect of supplementation on oxidative-mediated protein and DNA damage.

## Conclusion

Although TMM is well-known for its ethnomedicinal uses in curing many ailments and to improve respiratory health, but scientific evidences that supporting its therapeutic uses were limited to in vivo and in vitro models. Present work is the first to study the efficacy of TMM supplementation to improve respiratory health through a clinical approach. Findings revealed that TMM supplementation can effectively improve the respiratory health, immunity, as well as overall antioxidant status. Thus, suggesting TMM supplementation as a potential adjuvant therapy to the current drugs used for the management of respiratory diseases. Nonetheless, the lack of a placebo group is one important limitation in this study. Randomized controlled trial is recommended for future study to validate our findings. In addition, this study was not registered with any Clinical Trial Registry and will consider registering future studies.

## Methods

### Study design

This is an open-label, single-arm, prospective study that involved three-months period of supplementation. The study was conducted with full compliance to the principles of Helsinki Declaration, as well as criteria outlined in Malaysian Guidelines for Good Clinical Practice^75^. Eligibility was confirmed in accordance to protocol-checklist and written informed consent was obtained from each participant. The study was approved by principal investigator’s Institutional Ethics Committee (UCSI University, Malaysia, approval code IEC-2020-FMHS-026).

### Participants selection

This open-label study enrolled 50 volunteers (aged 30- to 50-year-old) with good general healthy condition. Recruitment was conducted in UCSI University, Kuala Lumpur, Malaysia. The following inclusion criteria were applied: (1) participants who were not undergoing any supplementation targeting respiratory health; (2) non-smoker; (3) participants who were able to fully understand protocol and study information given by investigators; and (4) participants who willing to give informed consent. Participants were exclude based on these exclusion criteria: (1) undergoing medication plan targeting respiratory system; (2) taking antimicrobial or antiviral medication during study entry; (3) undergone major surgical procedures within 6 months prior to study entry; and (4) pregnant or lactating woman. All participants were being provided with a participant information sheet and explained by investigator. A written informed consent was sought from each participant.

### Supplementation

At baseline visit, demographic characteristic and medical history of participants were collected. Participants were then started on three-months oral supplementation of TMM fine powder given in the form of capsule (TigerPro™, Nexus Wise, Selangor, Malaysia), at the dosage of 300 mg, twice daily. Three follow up visits were conducted with one month interval between each visit. A paper case report form (CRF) was used to capture the information of vital signs, pulmonary function, respiratory symptoms and self-evaluated severity of symptoms during each visit. Nasal lavage and saliva were also collected during each visit, at week 0, 4, 8 and 12 for laboratory investigation.

### Clinical assessment

Blood pressure and heart rate were measured using Omron automatic blood pressure monitor HEM 7120 (Omron Healthcare, Kyoto, Japan). Temperature was measured using Braun forehead infrared thermometer NTF 3000 (Braun GmbH, Kronberg, Germany). Pulmonary function was tested using spirometry method. Force Vital Capacity (FVC), Forced Expiratory Volume (FEV_1_) and FEV_1_/FVC ratio were assessed in triplicate using Contec SP70B handheld digital spirometer (Contec Medical Systems, Hebei, China). FVC (in litters) is the greatest total amount of air an individual can forcefully breathe out after breathing in as deeply as possible. FEV_1_ (in litters) is the amount of air an individual can force out of lungs in one second. It is a useful indicator for breathing problem. FEV_1_/FVC ratio was served as an indicator of general wellness of pulmonary function.

Validated nasal symptom questionnaire (NSQ) developed by Saito and his colleagues was being adopted with slight modification to evaluate participants sinonasal symptoms in this study^27^. NSQ is a self-administered questionnaire consisting of 10 items with 2 parts (I–II): (I) 8 items related to nasal symptoms and (II) 2 quality of life related items. Each item of the NSQ was divided into 6 levels: no symptoms at all (0 points); very mild (1 point) to very severe (5 pointes). Total points (NSQ score) (ranging from 0 to 50) were analysed. In addition, visual analogue scale (VAS) with the scale of 1 to 10 was being used to self-evaluate the severity of nasal symptoms.

### Laboratory examination

Nasal lavage was collected using NeilMed Sinus Rinse (NeilMed Pharmaceuticals, Inc., Santa Rosa, Canada), a low-pressure nasal irrigation tool with isotonic saline. Participant was required to bend forward their body to a comfort level and tilted their head down. Keeping their mouth open without holding breath, placed the cap snugly against nasal passage. Squeezed the bottle gently until the solution starts draining from the opposite nasal passage. The lavage fluids were collected using a 10 mL sterile tube. Lavage fluids were treated with N-acetyl cysteine in lab to disrupt mucus, supernatant was then collected after centrifugation for further analysis. The levels of the proinflammatory cytokines interleukin (IL)-1β and IL-8 were quantified in duplicate using BioLegend enzyme-linked immunosorbent assay (ELISA) kits (BioLegend CNS Inc., California, United States). Nasal lavage was collected because of its high reproducibility to assess upper respiratory tract inflammation, apart from its non-invasive and atraumatic nature of procedures^76^.

Unstimulated saliva samples were collected using a sterile 2.0-mL vial. Participants uncapped the vial, placed the straw into the vial, and passively drooled down the straw for 90s. All samples were assayed for different parameters in duplicate using respective kits. Immunoglobulin (IgA) was assayed using Elabsciecne QuicKey Human IgA ELISA Kit (Elabscience Biotechnology Co. Ltd, Texas, United States), total antioxidant capacity was assayed using Elabsciecne total antioxidant capacity (T-AOC) colorimetric assay kit (Elabscience Biotechnology Co. Ltd, Texas, United States), malondialdehyde (MDA) as lipid peroxidation biomarker was assayed using Elabsciecne MDA colorimetric assay Kit (Elabscience Biotechnology Co. Ltd, Texas, United States), 3-nitrotyrosine (3-NT) as biomarker of oxidative stress-derived protein damage was assayed using Elabsciecne 3-NT ELISA Kit (Elabscience Biotechnology Co. Ltd, Texas, United States) and 8-hydroxydeoxyguanosine (8-OHdG) as biomarker of oxidative stress-derived DNA damage was assayed using Elabsciecne 8-OHdG ELISA Kit (Elabscience Biotechnology Co. Ltd, Texas, United States). Key component that constitute the first line of immunological defence of respiratory tract is secretory IgA, and it is predominantly found in salivary secretions. Recent studies have suggested the potential role of salivary IgA as predictor of susceptibility to respiratory infections^77,78^. Levels of salivary oxidative stress were also found to be associated with the development of asthma^79^ and COPD^80^.

### Statistical analysis

Demographic characteristics were presented as categorical data, expressed in frequency and percentage. All outcomes were analysed as continuous dependent variables, presented as mean ± SD for normally distributed data or median (interquartile range) for non-normally distributed data. The changes in clinical and laboratory outcomes from baseline visit to last follow-up visit were analysed using general linear model (GLM) for repeated measures model. Within-subjects factor was defined as the sampling time point. Gender, age and BMI were tested as between-subject effect. Homogeneity of the variance and covariance structure of the dependent variables was assessed by Levene and Box M tests. Sphericity test of the residual covariance matrix was assessed using Mauchly’s sphericity test. Results considered significant if *p* < 0.05 with 95% of confidence interval. Statistical analysis was performed using SPSS 26.0 (IBM Corp., New York, United States) for MacOS.
